# CtrA activates the expression of glutathione S-transferase conferring oxidative stress resistance to *Ehrlichia chaffeensis*

**DOI:** 10.3389/fcimb.2022.1081614

**Published:** 2022-12-12

**Authors:** Qi’an Liang, Jiaqi Yan, Shuwen Zhang, Nan Yang, Meifang Li, Yongxin Jin, Fang Bai, Weihui Wu, Zhihui Cheng

**Affiliations:** Key Laboratory of Molecular Microbiology and Technology of the Ministry of Education, Department of Microbiology, College of Life Sciences, Nankai University, Tianjin, China

**Keywords:** *Ehrlichia chaffeensis*, human monocytic ehrlichiosis, oxidative stress, GST, CtrA

## Abstract

*Ehrlichia chaffeensis*, the causative agent of human monocytic ehrlichiosis (HME), is a Gram-negative obligatory intracellular bacterium, which infects and multiplies in human monocytes and macrophages. Host immune cells produce reactive oxygen species (ROS) to eliminate *E. chaffeensis* upon infection. *E. chaffeensis* global transcriptional regulator CtrA activates the expression of GshA and GshB to synthesize glutathione (GSH), the most potent natural antioxidant, upon oxidative stress to combat ROS damage. However, the mechanisms exploited by *E. chaffeensis* to utilize GSH are still unknown. Here, we found that in *E. chaffeensis* CtrA activated the expression of glutathione S-transferase (GST) upon oxidative stress, and *E. chaffeensis* GST utilizes GSH to eliminate ROS and confers the oxidative stress resistance to *E. chaffeensis*. We found that CtrA bound to the promoter regions of 211 genes, including *gst*, in *E. chaffeensis* using chromatin immunoprecipitation coupled to deep sequencing (ChIP-seq). Recombinant *E. chaffeensis* CtrA directly bound to the *gst* promoter region determined with electrophoretic mobility shift assay (EMSA), and activated the *gst* expression determined with reporter assay. Recombinant GST showed GSH conjugation activity towards its typical substrate 2,4-dinitrochlorobenzene (CDNB) *in vitro* and peptide nucleic acid (PNA) transfection of *E. chaffeensis*, which can knock down the *gst* transcription level, reduced bacterial survival upon oxidative stress. Our results demonstrate that *E. chaffeensis* CtrA regulates GSH utilization, which plays a critical role in resistance to oxidative stress, and aid in the development of new therapeutics for HME.

## Introduction

*Ehrlichia chaffeensis* is a Gram-negative obligatory intracellular bacterium that preferentially infects human monocytes or macrophages and causes human monocytic ehrlichiosis (HME). HME is one of the most prevalent, life-threatening emerging zoonoses with the clinical signs of headache, fever, myalgia, malaise accompanied by anemia, leukopenia, thrombocytopenia and the elevation of aminotransferases (Paddock and Childs, 2003; Kumagai et al., 2010). Nearly 40-60% of patients need hospitalization and the mortality is approximately 3% (Paddock and Childs, 2003).

CtrA is a response regulator of the two-component regulatory system CckA/CtrA and is conserved among α-proteobacteria including all sequenced members of the family *Anaplasmataceae*. The consensus CtrA-binding motifs are TTAA-N_7_-TTAAC (9-mer) and TTAACCAT (8-mer) (Quon et al., 1996; Laub et al., 2002). In *Caulobacter crescentus*, CtrA directly regulates at least 95 genes (Quon et al., 1996; Domian et al., 1997; Laub et al., 2002). In *E. chaffeensis*, CtrA binds to the promoter regions of *bolA*, *surE*, *ompA*, *gshA* and *gshB* and activates their expression (Cheng et al., 2011; Yan et al., 2022). All of these 5 genes are critical for *E. chaffeensis* infection and survival (Cheng et al., 2011; Mohan Kumar et al., 2013; Lina et al., 2016; Yan et al., 2022), but are not present in *C. crescentus* CtrA regulon, indicating that the adaptation of *E. chaffeensis* to its obligate intracellular growth has led to the emergence of unique CtrA regulon. However, the CtrA regulon in *E. chaffeensis* has not been fully illustrated.

Upon infection, host immune cells produce reactive oxygen species (ROS) to kill pathogens (Sharma et al., 2017; Sies and Jones, 2020). *E. chaffeensis* EtpE blocks the generation of ROS and Etf-1 upregulates host manganese superoxide dismutase (MnSOD) to reduce ROS levels in macrophages (Liu et al., 2012; Teymournejad and Rikihisa, 2020), but the production of ROS by neutrophils cannot be suppressed by *E. chaffeensis* (Lin and Rikihisa, 2007). The mechanisms exploited by *E. chaffeensis* to cope with the ROS produced by other cells remain largely unknown.

GshA and GshB are two enzymes that catalyze _L_-glutamate (_L_-Glu), _L_-cysteine (_L_-Cys) and _L_-glycine (_L_-Gly) to synthesize glutathione (GSH), the most potent natural antioxidant (**Figure S1**) (Smirnova and Oktyabrsky, 2005; Morris et al., 2013). In *E. chaffeensis* we found that the expression of *gshA* and *gshB* was activated by CtrA upon oxidative stress (Yan et al., 2022). In bacteria, GSH can be utilized by glutathione peroxidase (GPx) and glutathione S-transferase (GST) to eliminate ROS (Skopelitou et al., 2012a; Morris et al., 2013). However, *E. chaffeensis* lacks GPx (Rikihisa, 2015) and encodes only one GST (ECH_0847). The function and regulation of *E. chaffeensis* GST remain to be investigated. In this study, to gain insights into CtrA regulon in *E. chaffeensis* we performed chromatin immunoprecipitation coupled to deep sequencing (ChIP-seq) and identified that 211 genes, including *gst*, were regulated by CtrA. Then we characterized GST function by enzymatic assays *in vitro* and peptide nucleic acid (PNA) knockdown *in vivo*.

## Materials and methods

### Bacteria and cell culture

*E. chaffeensis* Arkansas strain was propagated in THP-1 cells in RPMI 1640 medium supplemented with 2 mM _L_-glutamine and 10% fetal bovine serum (FBS) (Every Green, Zhejiang, China) at 37°C in 5% CO_2_ and 95% air, as described previously (Cheng et al., 2006). The THP-1 cell line present in this study was obtained from Fuheng Biotechnology (Cat. number FH0112, Shanghai, China).

*Escherichia coli* strains (**Supplementary Table S1**) DH5α and BL21 (DE3) used for general cloning and protein expression, respectively, were cultured in LB broth supplemented with ampicillin (100 μg·mL^-1^), chloramphenicol (34 μg·mL^-1^) or kanamycin (50 µg·mL^-1^), as necessary.

### Expression and purification of recombinant proteins

The DNA fragment encoding full-length CtrA was cloned into pET-41a(+) to express recombinant CtrA (GST-rCtrA) as described previously (Cheng et al., 2006). The DNA fragments encoding GshA, GshB and GST were amplified using specific primers (**Supplementary Table S1**). The *gshA* fragment was cloned into pET-His-SUMO to express recombinant GshA (SUMO-rGshA) with an N-terminal His-tag. The fragment of *gshB* or *gst* was cloned into pET-33b(+) to express recombinant GshB (rGshB) or GST (rGST) with an N-terminal His-tag, respectively. The constructed plasmids were transformed into *E. coli* DH5α cells, extracted and confirmed by sequencing*. E. coli* BL21 (DE3) cells were transformed with the resulting plasmids to express SUMO-rGshA, rGshB and rGST respectively, with pET-41a(+) to express GST or pET-His-SUMO to express SUMO. *E*. *coli* BL21 (DE3) cells were induced to express recombinant proteins at an OD_600_ of 0.6 with 0.1 mM isopropyl-thio-β-D-galactoside (IPTG; Solarbio, Beijing, China) at 37°C for 4 h. All proteins were purified from soluble fraction with Ni-affinity chromatography and concentrated using an Amicon Ultra-0.5 Centrifugal Filter Unit (10 kDa MWCO).

### Chromatin immunoprecipitation coupled to deep sequencing (ChIP-seq)

Fixed cell sample was prepared from 2.5 × 10^7^ infected THP-1 cells (> 90% infected) following the instructions for suspension cell sample preparation from IGENEBOOK Biotechnology (Wuhan, China). The ChIP-seq experiments and data analyses were performed by IGENEBOOK Biotechnology. Briefly, cell culture was crosslinked with 1% (w/v) formaldehyde and incubated at room temperature for 10 min. Crosslinking was terminated by adding 125 mM _L_-Gly. Crosslinked cells were washed twice with 1 × PBS at 4°C, pelleted, frozen in liquid nitrogen and subjected to ChIP-seq. Rabbit antiserum against *E. chaffeensis* CtrA (Yan et al., 2022) was used for immunoprecipitation of *E. chaffeensis* chromosomal DNA fragments bound by CtrA. Total DNA before immunoprecipitation was used as input DNA. Fold enrichment was expressed as immunoprecipitation signal normalized against input signal.

### Electrophoresis mobility shift assay

Electrophoresis mobility shift assay (EMSA) was performed as described previously (Cheng et al., 2011). Briefly, the promoter regions of *gst* (305 bp) and *p28* (260 bp) were amplified with PCR using specific primers (**Supplementary Table S1**). Fifty picomoles of purified GST-rCtrA or GST (negative control) was incubated with 50 ng of DNA probes in a 20-μL reaction mixture containing 10 mM Tris-HCl (pH 7.5), 1 mM DTT and 1% (w/v) _L_-Gly on ice for 30 min. Samples were loaded onto 8% native polyacrylamide gel in 1 × TBE buffer [89 mM Tris (pH 8.3), 89 mM boric acid and 2 mM EDTA], which had been prerun at 100 V for 1 h, and electrophoresed at 10 mA on ice for 1.5 h. The gel was stained in 1 × TBE containing 0.5 μg·mL^-1^ ethidium bromide at room temperature for 10 min. Bands were visualized using a ChemiDoc XRS+ molecular imager (Bio-Rad).

### Construction of enhanced green fluorescent protein fusions and reporter assay

Enhanced green fluorescent protein (EGFP) fusions were constructed as described previously (Duan et al., 2021). Briefly, the promoter region of *gst* or *p28* was amplified and inserted upstream of the promoter-less *egfp* gene in a pQE60 vector. *E. coli* BL21 (DE3) cells containing pACYCDuet-1 harboring *ctrA* (pACYCDuet-1-rCtrA) or the empty pACYCDuet-1 vector were transformed with the pQE60-promoter-EGFP fusion constructs. After induction of rCtrA expression with 0.05 mM IPTG at 37°C for 3 h, bacterial samples were collected and subjected to Western blotting to measure the amounts of EGFP, RpoA, and rCtrA in each sample.

### Western blotting

Western blotting was performed as described previously (Yan et al., 2022). Briefly, to detect the amount of EGFP in the reporter assay, the same numbers of *E. coli* cells were collected by centrifugation at 12000 × *g* for 1 min, resuspended in 1 × SDS sample buffer [62.5 mM Tris-HCl (pH 6.8), 2% (w/v) SDS, 0.02% (w/v) bromophenol blue, 1% (v/v) β-mercaptoethanol and 10% (v/v) glycerol] and boiled for 10 min. The samples were then subjected to 12% SDS-polyacrylamide gel electrophoresis (SDS-PAGE) and transferred to a PVDF membrane (Millipore, Co. Cork, Ireland). The protein levels of EGFP, RpoA, and rCtrA were determined using a mouse monoclonal anti-GFP antibody (GeneTex Inc., North America, GTX628528), mouse monoclonal anti-*E. coli* RNA polymerase α antibody (BioLegend, San Diego, CA, United States, 663104), and rabbit antiserum against *E. chaffeensis* CtrA (Yan et al., 2022), respectively. The relative amounts of EGFP or RpoA in *E. coli* BL21 (DE3) cells expressing rCtrA were normalized against those in *E. coli* BL21 (DE3) cells containing pACYCDuet-1 vector.

### Isolation of host cell-free *E. chaffeensis*

*E. chaffeensis* was isolated from infected THP-1 cells as described previously (Yan et al., 2022). Briefly, *E. chaffeensis*-infected THP-1 cells (2 × 10^7^ cells, > 90% infected) were harvested at 600 × *g* at room temperature for 5 min. The pellet was suspended in fresh culture medium and passed through a 23-gauge needle with a syringe on ice for 20 times to rupture the host cell membrane. To remove unbroken cells and cell debris, the mixture was centrifuged at 1,000 × *g* at 4°C for 5 min. Bacteria in supernatant were collected by additional centrifugation at 10,000 × *g* at 4°C for 10 min. The bacterial pellet was suspended in fresh culture medium for synchronous culture or in 0.3 M sucrose for PNA transfection.

### Quantitative RT-PCR

Total RNA was extracted from each sample and reverse transcribed to cDNA as described previously (Duan et al., 2021). The amounts of *E. chaffeensis* 16S rRNA, *ctrA*, *gst, gshA*, *gshB*, and human *GAPDH* were determined with qRT-PCR using specific primers (**Supplementary Table S1**) and the ChamQ Universal SYBR qPCR Master Mix (Vazyme, Nanjing, China) on a StepOnePlus Real-Time PCR System (Applied Biosystems, MA, USA). The expression levels of bacterial genes were normalized against that of *E. chaffeensis* 16S rRNA. Relative bacterial number was determined as the amount of bacterial 16S rRNA normalized against that of human *GAPDH* mRNA.

### Activity assay of glutathione S-transferase

The activity of rGST was determined as described previously with minor modifications (Habig and Jakoby, 1981). Briefly, the reaction mixture was composed of 100 mM Na_3_PO_4_ (pH 6.5), 1 mM 2,4-dinitrochlorobenzene (CDNB; Xiya Reagent, Shandong, China) and varying concentrations of GSH. Reactions were initiated by adding 3 μg of purified rGST or SUMO as a negative control in a final volume of 200 μL, then incubated at 37°C for 30 min. The formation of GSH-CDNB conjugate was measured by OD_340_.

### Activity assay of glutamate-cysteine ligase

The activity of SUMO-rGshA was determined as described previously with minor modifications (Tu and Anders, 1998). Briefly, the reaction mixture was composed of 100 mM Tris-HCl (pH 8.0), 50 mM KCl, 20 mM MgCl_2_, 2 mM EDTA, 5 mM ATP, 10 mM _L_-α-aminobutyric acid and varying concentrations of monosodium _L_-glutamate. Reactions were initiated by adding 1.25 μg of purified SUMO-rGshA or SUMO as a negative control in a final volume of 80 μL, then incubated at 37°C for 30 min. Twenty microliters of 5% (w/v) SDS were added into each reaction mixture to terminate the reaction and the concentration of inorganic phosphate (P_i_) generated by the reaction (**Figure S1**) was determined as described previously with minor modifications (Satoh et al., 2006). Briefly, 100 μL of reagent mixture containing 24 mg·mL^-1^ ascorbic acid in (NH_4_)_2_MoO_4_-H_2_SO_4_ solution [1.145 g (NH_4_)_2_MoO_4_ and 5.95 mL H_2_SO_4_ in 250 mL of water] was added to each reaction mixture. Reaction mixtures were incubated at room temperature for 2 h and concentrations of P_i_ were measured by OD_750_ using Varioskan Flash (Thermo Fisher Scientific). To calibrate the P_i_ concentration, 80 μL of increasing concentrations of KH_2_PO_4_ from 0 to 1,000 μM was mixed with 20 μL 5% (w/v) SDS and subjected to P_i_ determination as described above to generate a standard curve for each experiment.

### Activity assay of glutathione synthetase

The activity of rGshB was determined as described previously with minor modifications (Oppenheimer et al., 1979). Briefly, the reaction mixture was composed of 100 mM Tris-HCl (pH 8.2), 50 mM KCl, 20 mM MgCl_2_, 2 mM EDTA, 10 mM Na_3_ATP, 2.5 mM DTT, 5 mM _L_-γ-_L_-glutamyl-_L_-cysteine (γ-GC) and varying concentrations of _L_-Gly. Reactions were initiated by adding 0.75 μg of purified rGshB or SUMO as a negative control in a final volume of 80 μL, then incubated at 37°C for 30 min. The concentration of P_i_ generated by these reactions (**Figure S1**) was measured as described above.

### PNA transfection

PNA transfection to knock down CtrA, GST, GshA or GshB in *E. chaffeensis* was performed as described previously (Yan et al., 2021). Antisense PNA oligomers targeting 31-46 bp following the start codon of *ctrA* (CtrA PNA), 10-23 bp following the start codon of *gst* (GST PNA), 13-28 bp following the start codon of *gshA* (GshA PNA) and 19-34 bp following the start codon of *gshB* (GshB PNA) and a control PNA with a scrambled sequence (CTL PNA) were synthesized by KareBay Biochem, Inc. (Ningbo, China; **Supplementary Table S1**). The knockdown efficiency of CtrA PNA has been verified previously (Yan et al., 2022). To verify the specific binding of PNAs to their targets, ssRNAs derived from *gst* (GST ssRNA, 3-33 bp following the start codon of *gst* mRNA), *gshA* (GshA ssRNA, 6-37 bp following the start codon of *gshA* mRNA) and *gshB* (GshB ssRNA, 12-43 bp following the start codon of *gshB* mRNA) were synthesized by Genewiz (Suzhou, China; **Supplementary Table S1**). Hybridization of ssRNA with PNA oligomer was performed as described with minor modifications (Yan et al., 2018). Briefly, 80 pmol of ssRNA and 80 pmol of gene-specific PNA or CTL PNA were added into hybridization buffer containing 250 mM Tris-HCl (pH 7.2) in a final volume of 15 μL. Hybridization was performed on a BIO-GENER GE4852T thermocycler (Hangzhou, China) with the following program: 95°C for 3 min, then gradual cooling to 20°C over 75 cycles (20 s per cycle), 20°C for 1 min. Hybridization mixtures were then subjected to 12% native polyacrylamide gel electrophoresis and bands were visualized by GelGreen nucleic acid staining (Biomed, Beijing, China).

Three micrograms of CtrA PNA, GST PNA, GshA PNA, GshB PNA or CTL PNA dissolved in nuclease-free water were mixed with 100 μL of host cell-free *E. chaffeensis* in 0.3 M sucrose, then incubated on ice for 15 min. Electroporation was conducted at 2,000 V, 25 μF, and 400 Ω with a 10-ms pulse using a Gene Pulser Xcell electroporation system (Bio-Rad, Hercules, CA, United States) in a 2-mm electroporation cuvette (Bio-Rad). Then the PNA-transfected *E. chaffeensis* was transferred to a T25 flask to infect 5 × 10^5^ THP-1 cells and incubated at 37°C for 2 h with gentle shaking every 15 min to facilitate bacterial internalization. To detect the effect of GST PNA, GshA PNA and GshB PNA transfection, infected cells were harvested at 36 hours post infection (h p.i.). The expression levels of *gst*, *gshA* and *gshB* were determined using qRT-PCR.

### H_2_O_2_ assay

*E. chaffeensis* was treated with H_2_O_2_ as described previously (Yen et al., 2020). THP-1 cells were synchronously infected with CtrA PNA-, GST PNA-, GshA PNA-, GshB PNA- or CTL PNA-transfected *E. chaffeensis*. At 36 h p.i., 20 μL of 10 mM H_2_O_2_ diluted in culture medium was added into 2 mL of the synchronous culture, reaching a final concentration of 100 μM. After 2 h of treatment at 37°C, the bacterial number in each sample was determined with qRT-PCR.

### Statistical analysis

CtrA ChIP-seq assay was performed for a single sample and ChIP-seq data were analyzed by IGENEBOOK Biotechnology (Wuhan, China). All other experiments were performed at least three times. Statistical analyses were performed using GraphPad Prism 7.0. Statistical significance of a two-group comparison was assessed using Student’s *t* test (two-tailed). A value of *P* < 0.05 was considered significant.

## Results

### The promoter regions of 211 genes including *gst* are enriched in CtrA ChIP-seq assay using antiserum against *E. chaffeensis* CtrA

**Figure 1:**
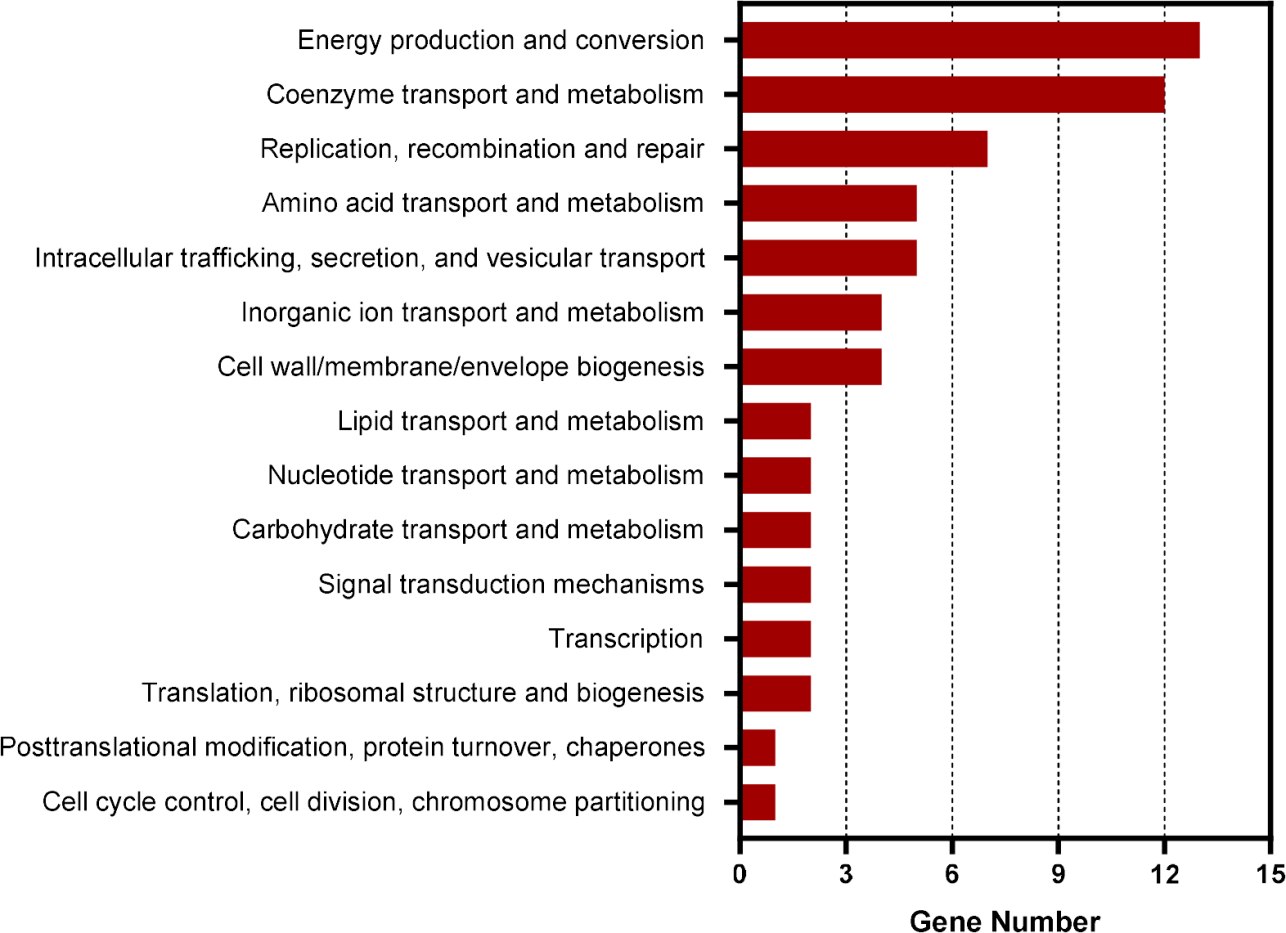
COG analysis of the promoter regions of genes enriched in CtrA ChIP-seq assay. COG analysis was accomplished by COGClassifier (https://github.com/moshi4/COGclassifier). The length of bar indicates the number of genes.

To identify the genes regulated by CtrA in *E. chaffeensis*, we first performed ChIP-seq assay. Using rabbit antiserum against *E. chaffeensis* CtrA, we identified that the promoter regions of 211 genes were enriched by more than 1.5-fold (*P*-value < 0.01) compared to input DNA (**Supplementary Table S2**). Among these genes, 69 were annotated and 64 were successfully classified into Cluster of Orthologous Group (COG) functional categories (*E*-value < 0.01). COG analysis revealed that these genes were involved in coenzyme transport and metabolism, energy production and conversion, replication, recombination, and repair, and various other functions (**Figure 1**). Consistent with the previous studies (Cheng et al., 2011; Yan et al., 2022), the promoter regions of *ctrA, bolA*, *surE* and *gshB* were enriched by 1.64-, 1.72-, 1.61- and 1.71-fold, respectively. The promoter regions of *gshA* and *ompA* were also enriched by 1.45- and 1.30-fold respectively (**Supplementary table S2**).

Among these enriched promoter regions, notably, the *gst* promoter region was enriched 1.81-fold compared to input DNA (**Supplementary Table S2**). GST is an enzyme that is able to catalyze the reaction of GSH with ROS, then eliminate ROS (Skopelitou et al., 2012a). The amino acid sequence of *E. chaffeensis* GST showed 34.35% identity with that of *Agrobacterium tumefaciens* GST (GenBank ID: AAK86108.1) (**Figure S2A**) (Skopelitou et al., 2012b). We found a 9-mer CtrA binding motif (TTAA-N_7_-TTAAC) in the *gst* promoter region (-181 to -166 calculated from the translational start codon) using pDRAW32 DNA analysis software (http://acaclone.com/) (**Figure S2B**). These results suggest that CtrA regulates the *gst* expression in *E. chaffeensis*.

### CtrA activates the *gst* expression in *E. chaffeensis* upon oxidative stress

**Figure 2:**
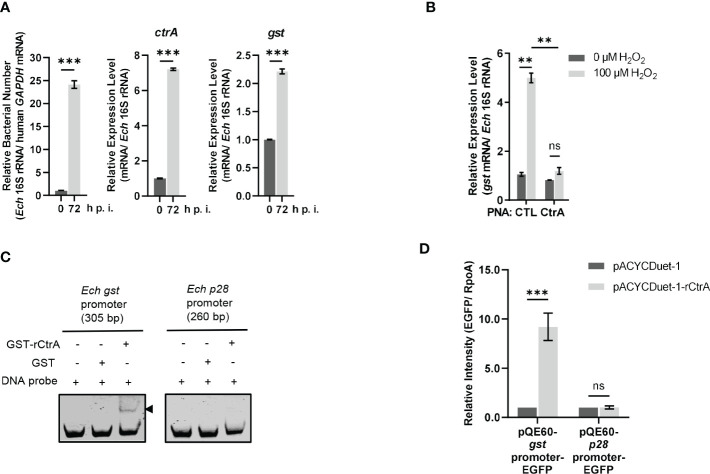
CtrA binds to the *gst* promoter region and activates the *gst* expression upon oxidative stress. **(A)** The expression levels of *ctrA* and *gst* and relative bacterial numbers at different stages of *E. chaffeensis* intracellular growth. RNA samples were prepared from synchronously cultured *E. chaffeensis* in THP-1 cells at 0 h and 72 h p.i. The expression level of each gene was determined with qRT-PCR and normalized against that of *E. chaffeensis* 16S rRNA. Relative bacterial number was determined as the amount of bacterial 16S rRNA normalized against that of human *GAPDH* mRNA. Relative values to the amount at 0 h p.i. are shown. Data indicate means ± standard deviations (*n* = 3). The significant differences are represented by *P*-values determined with Student’s *t* test (*** indicates *P* < 0.001). **(B)** CtrA activates the *gst* expression in *E. chaffeensis* upon oxidative stress. At 36 h p.i. THP-1 cells synchronously infected with CTL PNA- or CtrA PNA-transfected *E. chaffeensis* were treated with H_2_O_2_ at a final concentration of 100 μM or culture medium (CTL) at 37°C for 2 h. The *gst* expression level was determined with qRT-PCR and normalized against that of *E. chaffeensis* 16S rRNA. Relative values to the *gst* amount of CTL PNA-transfected *E. chaffeensis* without H_2_O_2_ treatment are shown. Data indicate means ± standard deviations (n = 3). The significant differences are represented by *P*-values determined with Student’s *t* test (ns indicates *P* > 0.05, and ** indicates *P* < 0.01). **(C)** CtrA binds to the *gst* promoter. DNA probe (50 ng) was incubated alone, with GST (2.5 µM) or with GST-rCtrA (2.5 µM) on ice for 30 min. Shifted band is indicated by an arrowhead. The *p28* promoter region is shown as a negative control. The length (bp) of the probe is shown above each panel. **(D)** CtrA activates the *gst* expression. *E. coli* BL21 (DE3) cells grown to an OD_600_ of 0.4 were induced to express rCtrA with 0.05 mM IPTG at 37 °C for 3 h. The amount of EGFP or RpoA was determined using Western blotting. The relative intensities of EGFP to those of RpoA were measured by Image J and calculated by setting the value of bacteria containing pACYCDuet-1 and corresponding pQE60-promoter-EGFP fusion construct as 1. Data indicate means ± standard deviations (*n* = 3). The significant differences are represented by *P*-values determined with Student’s *t* test (ns indicates *P* > 0.05, and *** indicates *P* < 0.001).

We then investigated whether the *gst* expression was regulated by CtrA. CtrA is expressed at the late stage of *E. chaffeensis* intracellular growth (Cheng et al., 2011; Yan et al., 2022). Using qRT-PCR, we examined the *gst* expression pattern in synchronously cultured *E. chaffeensis* in THP-1 cells. We found that the *gst* expression was also upregulated at the late stage of *E. chaffeensis* intracellular growth (**Figure 2A**), indicating that the *gst* expression may be regulated by CtrA.

GshA and GshB are glutamate-cysteine ligase and glutathione synthetase respectively that catalyze GSH synthesis from _L_-Glu, _L_-Gly, _L_-Cys and ATP (**Figure S1**). Previously we found that CtrA activates the expression of *gshA* and *gshB* in *E. chaffeensis* upon oxidative stress (Yan et al., 2022). We then investigated whether the *gst* expression was activated by CtrA upon oxidative stress. PNA is a DNA mimic that has been shown to bind single- and double-stranded DNA and RNA with high affinity and specificity (Pelc et al., 2015), and can be used to knock down the levels of *E. chaffeensis* proteins (Sharma et al., 2017; Yan et al., 2018; Yan et al., 2021; Yan et al., 2022). Previously we used PNA to specifically knock down CtrA level in *E. chaffeensis* (Yan et al., 2022). Host cell-free *E. chaffeensis* was transfected with CtrA PNA or CTL PNA, then used to infect THP-1 cells. At 36 h p.i. the infected THP-1 cells were treated with 100 μM H_2_O_2_ at 37°C for 2 h. We found that the *gst* expression in *E. chaffeensis* transfected with CTL PNA was significantly upregulated after H_2_O_2_ treatment, suggesting that GST may be required for conferring oxidative stress resistance to *E. chaffeensis* (**Figure 2B**). After CtrA level was knocked down by CtrA PNA, the *gst* expression showed no changes after H_2_O_2_ treatment (**Figure 2B**), indicating that the *gst* expression upon oxidative stress is activated by CtrA.

We then performed EMSA using GST-rCtrA to investigate whether CtrA directly binds to the *gst* promoter. Purified GST-rCtrA showed a single band on the SDS-PAGE gel (**Figure S3**). The DNA probe derived from the *gst* promoter region shifted upon incubation with GST-rCtrA (**Figure 2C**). The binding specificity was confirmed by the result that no shifted band was detected when the DNA probe was incubated with purified GST protein or when a DNA probe derived from the *p28* promoter region, which is not bound by CtrA, was incubated with GST-rCtrA (**Figure 2C**). These results indicate that CtrA directly binds to the *gst* promoter in *E. chaffeensis*.

Due to the lack of effective genetic manipulation methods in *E. chaffeensis*, in order to investigate whether CtrA activates the *gst* expression, we used an *E. coli* reporter assay system (Cheng et al., 2011; Duan et al., 2021; Yan et al., 2022). The *gst* promoter region was inserted upstream of the promoter-less *egfp* gene in the pQE60 plasmid to generate *gst* promoter-EGFP fusion construct. The *p28* promoter region was used to generate *p28* promoter-EGFP fusion construct as a negative control. *E. coli* BL21 (DE3) cells containing pACYCDuet-1 vector harboring *E. chaffeensis ctrA* gene (pACYCDuet-1-rCtrA) or pACYCDuet-1 vector only (negative control) were transformed with the EGFP fusion constructs, respectively. The rCtrA expression induced by IPTG resulted in a significantly higher expression of EGFP in bacteria harboring *gst* promoter-EGFP construct compared to the vector control, while it had no effect on bacteria harboring *p28* promoter-EGFP construct (**Figure 2D**; **Figure S4**). These results indicate that CtrA activates the *gst* expression in *E. chaffeensis.*

### *E. chaffeensis* GST, GshA and GshB are functional *in vitro*

**Figure 3:**
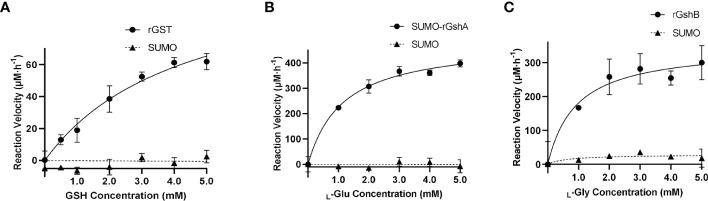
*E. chaffeensis* GST, GshA and GshB are functional *in vitro*. **(A)** Michaelis-Menten plot of rGST reaction velocity versus GSH concentration. **(B)** Michaelis-Menten plot of SUMO-rGshA reaction velocity versus _L_-Glu concentration. **(C)** Michaelis-Menten plot of rGshB reaction velocity versus _L_-Gly concentration.

We next determined the GST function *in vitro*. The purified rGST and the negative control SUMO, which was purified using the same method, showed single bands on the SDS-PAGE gel (**Figure S5**). The enzymatic activity of rGST was measured by GSH-CDNB conjugate formation (**Figure 3A**). The *K*_m_ value of rGST was determined for GSH. The *K*_m_ and *V*_max_ values of the enzymes are listed in **Table 1**. The results showed that *E. chaffeensis* rGST was functional and its *K*_m_ value for GSH was found to be in a similar range with that of *A. tumefaciens* GST reported previously (Skopelitou et al., 2012a), indicating that GST is a functional enzyme in *E. chaffeensis* that catalyzes the conjugation of GSH to its typical substrate CDNB.

**Table 1:** *K*_m_ and *V*_max_ of rGST, SUMO-rGshA and rGshB.

Enzyme	*K*_m_ (mM)	*V*_max_ (μmol·min^-1^·mg^-1^)
rGST	4.72 ± 1.33 (GSH)	0.14 ± 0.02
SUMO-rGshA	1.03 ± 0.02 (_L-_Glu)	0.52 ± 0.02
rGshB	0.94 ± 0.32 (_L-_Gly)	0.63 ± 0.11

In our previous study, we verified the functions of *E. chaffeensis* GshA and GshB by complementing *Pseudomonas aeruginosa* respective mutants (Yan et al., 2022). Here, we also determined the functions of *E. chaffeensis* GshA and GshB *in vitro*. Since rGshA was unable to be expressed efficiently in *E. coli* BL21 (DE3) cells, we constructed a plasmid to express SUMO-fused recombinant GshA (SUMO-rGshA). The purified SUMO-rGshA and rGshB showed single bands on the SDS-PAGE gel (**Figure S5**). The enzymatic activities of SUMO-rGshA and rGshB were measured based on P_i_ formation (**Figures 3B, C**). The *K*_m_ value of SUMO-rGshA or rGshB was determined for _L_-Glu or _L_-Gly respectively. The *K*_m_ and *V*_max_ of the enzymes are listed in **Table 1**. The results showed that SUMO-rGshA was functional and the *K*_m_ value for _L_-Glu was found to be in a similar range with that of *A. tumefaciens* GshA (Gromes et al., 2008). Also, rGshB was functional and the *K*_m_ value for _L_-Gly was found to be in a similar range with that of *E. coli* GshB (Tanaka et al., 1997). These results indicate that GshA and GshB are functional enzymes in *E. chaffeensis*, which catalyze the synthesis of GSH.

### PNA targeting *gst*, *gshA* or *gshB* reduces the survival ability of *E. chaffeensis* upon oxidative stress

**Figure 4:**
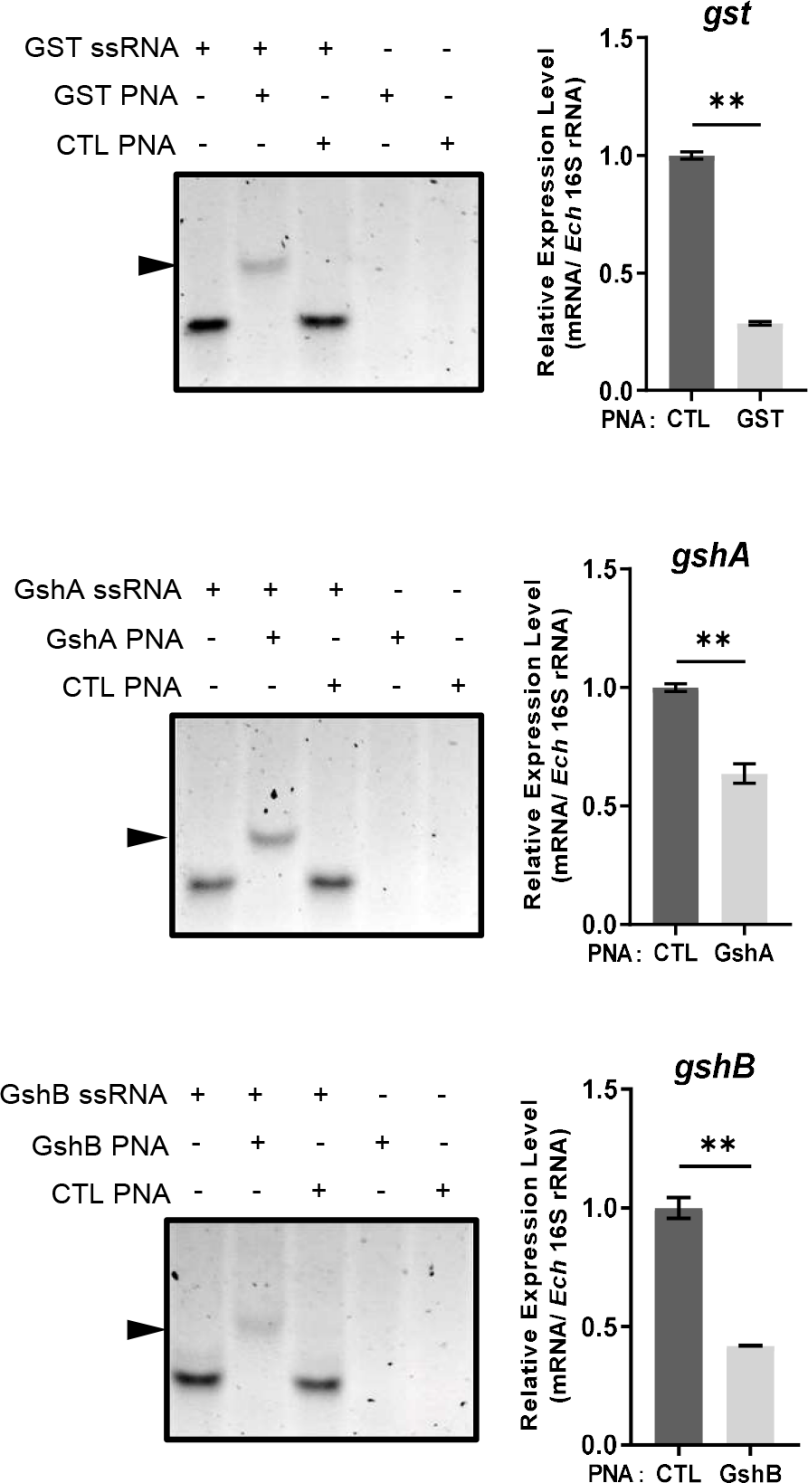
PNA transfection inhibits the expression of *gst*, *gshA*, and *gshB* respectively. GST PNA, GshA PNA or GshB PNA binds to its corresponding ssRNA (left). ssRNA probe (80 pmol) alone, with its corresponding PNA (80 pmol) or with CTL PNA (80 pmol) was annealed from 95 °C to 20 °C. The same amount of corresponding PNA (80 pmol) or CTL PNA (80 pmol) alone was treated at the same condition. Shifted bands are indicated by arrows. The expression levels of *gst*, *gshA* and *gshB* were determined with qRT-PCR and normalized against that of *E. chaffeensis* 16S rRNA (right). Relative values to the amount of CTL PNA-transfected *E. chaffeensis* are shown. Data indicate means ± standard deviations (*n* = 3). The significant differences are represented by *P*-values determined with Student’s *t* test (** indicates *P* < 0.01).

**Figure 5:**
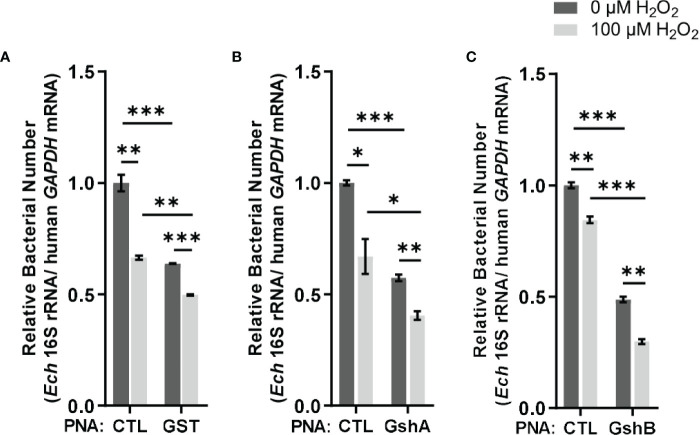
Transfection of GST PNA **(A)**, GshA PNA **(B)** or GshB PNA **(C)** reduces the survival ability of *E. chaffeensis* upon oxidative stress. At 36 h p.i. THP-1 cells synchronously infected with GST PNA-, GshA PNA-, GshB PNA- or CTL PNA-transfected *E. chaffeensis* were treated with H_2_O_2_ at a final concentration of 100 μM or culture medium (CTL) at 37 °C for 2 h. Relative bacterial numbers was determined as the amount of bacterial 16S rRNA normalized against that of human *GAPDH* mRNA. Relative values to the amount of CTL PNA-transfected *E. chaffeensis* without H_2_O_2_ treatment are shown. Data indicate means ± standard deviations (*n* = 3). The significant differences are represented by *P*-values determined with Student’s *t* test (* indicates *P* < 0.05, ** indicates *P* < 0.01, and *** indicates *P* < 0.001).

Since *E. chaffeensis* GST was functional *in vitro*, we then investigated its function *in vivo*. We designed GST PNA that specifically binds to the region following the translation start codon of *gst* and confirmed the specificity by showing that GST PNA bound to the GST ssRNA (synthesized *gst* mRNA fragment, 3-33 bp following the start codon) (**Figure 4**). Transfection of host cell-free *E. chaffeensis* with GST PNA significantly reduced the *gst* mRNA level at 36 h p.i. (**Figure 4**). We then investigated whether the reduction of the *gst* expression influenced the bacterial survival upon oxidative stress. At 36 h p.i. the infected THP-1 cells were treated with 100 μM H_2_O_2_ at 37 °C for 2 h. The survival ability of *E. chaffeensis* transfected with GST PNA was significantly lower than that of *E. chaffeensis* transfected CTL PNA with H_2_O_2_ treatment (**Figure 5A**), indicating that GST is critical for *E. chaffeensis* to cope with oxidative stress.

We also determined the survival ability of *E. chaffeensis* transfected with GshA PNA or GshB PNA upon oxidative stress. We designed GshA PNA and GshB PNA and confirmed the specificity by showing that both GshA PNA and GshB PNA bound to their corresponding ssRNA (synthesized *gshA* or *gshB* mRNA fragment, 6-37 bp or 12-43 bp following the start codon, respectively) (**Figure 4**). Transfection of host cell-free *E. chaffeensis* with GshA PNA or GshB PNA significantly reduced the mRNA level of *gshA* or *gshB* at 36 h p.i. (**Figure 4**). At 36 h p.i. the infected THP-1 cells were treated with H_2_O_2_ as described above. The survival ability of *E. chaffeensis* transfected with GshA PNA or GshB PNA was also significantly lower than that of *E. chaffeensis* transfected CTL PNA (**Figures 5B, C**). All these results together indicate that GSH synthesis and utilization are critical for *E. chaffeensis* to cope with oxidative stress.

## Discussion

Due to the reduction of bacterial genome during evolution, *E. chaffeensis* only encodes a few transcriptional regulators (Cheng et al., 2015), which leads to merging of genes, especially genes critical for bacterial infection and survival, under the control of these regulators. We found the number of genes regulated by CtrA in *E. chaffeensis* was higher than that in *C. crescentus*. Previously it has been reported that CtrA regulates the expression of *bolA*, *ompA*, *surE*, *gshA* and *gshB* in *E. chaffeensis* (Cheng et al., 2011; Yan et al., 2022). Here we found that CtrA regulated the *gst* expression in *E. chaffeensis*. All these genes are critical for *E. chaffeensis* infection and survival (Cheng et al., 2011; Yan et al., 2022), but are not present in CtrA regulons in other bacteria. Thus, identification of CtrA regulon in *E. chaffeensis* can provide new information on the mechanisms exploited by *E. chaffeensis* to find its unique niche during infection. Using ChIP-seq, we identified 211 genes bound by CtrA. Among these genes, the annotated genes belong to 15 categories analyzed by COG and may contribute to *E*. *chaffeensis* virulence. For example, HupB is a nucleoid-associated protein that promotes the survival of *Mycobacterium tuberculosis* in iron-limited host cell environment by inducing the biosynthesis of mycobactin, an Fe(III)-specific high affinity siderophore (Pandey et al., 2014; Kriel et al., 2018). TatC is a component of the twin-arginine translocation (Tat) system, which is predicted to export virulence factors from cytoplasm into periplasm in *Anaplasma phagocytophilum* (Dugat et al., 2014) and is essential for the viability of *M*. *tuberculosis* (De Buck et al., 2008). SecF and SecG are components of the Sec translocation system, which may participate in the type IV secretion system assembly in *A. phagocytophilum* (Dugat et al., 2014). PstC is a component of the phosphate-specific transport (Pst) system involved in phosphate uptake to support bacterial growth in *E*. *coli* (Hsieh and Wanner, 2010). The roles of these genes during *E. chaffeensis* infection remain to be investigated.

GSTs are enzymes that conjugate GSH to various substrates for ROS elimination and detoxification (Skopelitou et al., 2012a; Skopelitou et al., 2012b). *E. chaffeensis* lacks GPx, and encodes only one GST, unlike that multiple GSTs present in other bacteria. Here, we showed that *E. chaffeensis* GST played a critical role in GSH-dependent ROS elimination. After interaction with ROS, GSH is converted to oxidized GSH (GSSG). While GSSG is unable to be recycled back to GSH due to the lack of glutathione reductase (GR) in *E. chaffeensis* (Rikihisa, 2015), other mechanisms may be involved in GSSG processing. In *Novosphingobium aromaticivorans*, an Atm1/ABCB7/HMT1/ABCB6 family ATP-binding cassette (ABC) exporter, *Na*Atm1, is able to export various GSH derivatives, including GSSG, out of bacteria (Lee et al., 2014). In *E. chaffeensis*, ECH_0313, a putative ABC transporter, shows 38% sequence identity with *Na*Atm1. It is likely that *E. chaffeensis* exports GSSG *via* an ABC exporter.

Previously we found that CtrA activates the expression of *gshA* and *gshB*, which encode enzymes for GSH synthesis, upon oxidative stress (Yan et al., 2022). Here we found that CtrA activated the *gst* expression upon oxidative stress. Interestingly, the ChIP-seq results showed that the promoter region of ECH_0313 was bound by CtrA. Together, these results indicate that in *E. chaffeensis* CtrA regulate the GSH synthesis and utilization pathway to eliminate ROS and maintain redox homeostasis. However, the mechanism by which oxidative stress signal is transmitted to CtrA is still unknown. CtrA activation is regulated by the kinase/phosphatase activity of CckA, the cognate histidine kinase of CtrA. In *C. crescentus*, the kinase/phosphatase activity of CckA is regulated by the interaction with DivK and DivL or the binding of its PAS domain to cyclic-di-GMP (Iniesta et al., 2010; Tsokos et al., 2011; Mann et al., 2016). In *E. chaffeensis*, DivK and DivL are missing and CckA lacks PAS domains (Cheng et al., 2006; Cheng et al., 2011). Thus, the kinase/phosphatase activity of CckA may be controlled by other mechanisms. CckA may detect the redox condition or the concentration of GSSG directly, or indirectly through other pathways such as two other two-component regulatory systems, NtrY/NtrX and PleC/PleD (Cheng et al., 2006; Kumagai et al., 2006).

The lack of effective genetic manipulation methods greatly hampered the function research of *E. chaffeensis* genes (Cheng et al., 2013). In the present study, we characterized *E. chaffeensis* GST function using purified recombinant protein *in vitro* and PNA to temporarily knock down the *gst* expression *in vivo*. Using the same methods, we also characterized *E. chaffeensis* GshA and GshB, which suggests that a combination of these two methods can be used to study the function and pathogenesis of *E. chaffeensis* proteins.

## Data availability statement

CtrA ChIP-seq raw data have been deposited in the NCBI Short Read Archive (SRA) database (https://www.ncbi.nlm.nih.gov/sra) under the accession number PRJNA898263. The original data presented in the study are included in the article/**Supplementary Material**, further inquiries can be directed to the corresponding author.

## Author contributions

QL, JY and ZC conceived, designed the experiments, and wrote the manuscript. QL, JY, SZ, NY and ML performed the experiments. YJ, FB and WW analyzed the data. All authors contributed to the article and approved the submitted version.

## Supplementary material

Supplementary Table S1 Bacterial strains, plasmids, primers, PNAs and ssRNAs used in this study.

Supplementary Table S2
*E. chaffeensis* CtrA ChIP-seq results.
