# Supplementary figures and images for: CtrA activates the expression of glutathione S-transferase conferring oxidative stress resistance to Ehrlichia chaffeensis
Source: Front Cell Infect Microbiol. 2022 Dec 12;12:1081614. doi: 10.3389/fcimb.2022.1081614 (PMC9791040; doi:10.3389/fcimb.2022.1081614)

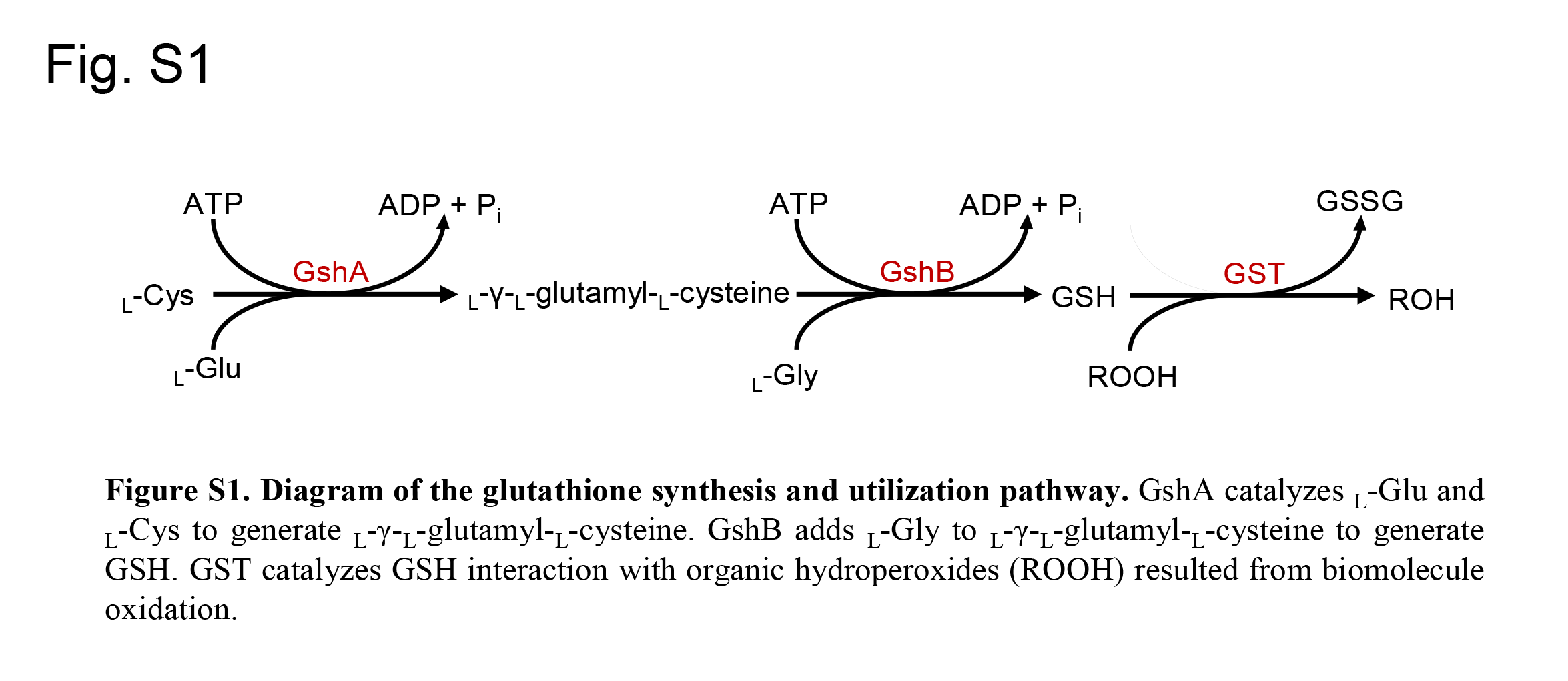

Supplement: Supplementary file 1 [file Image_1.tif]

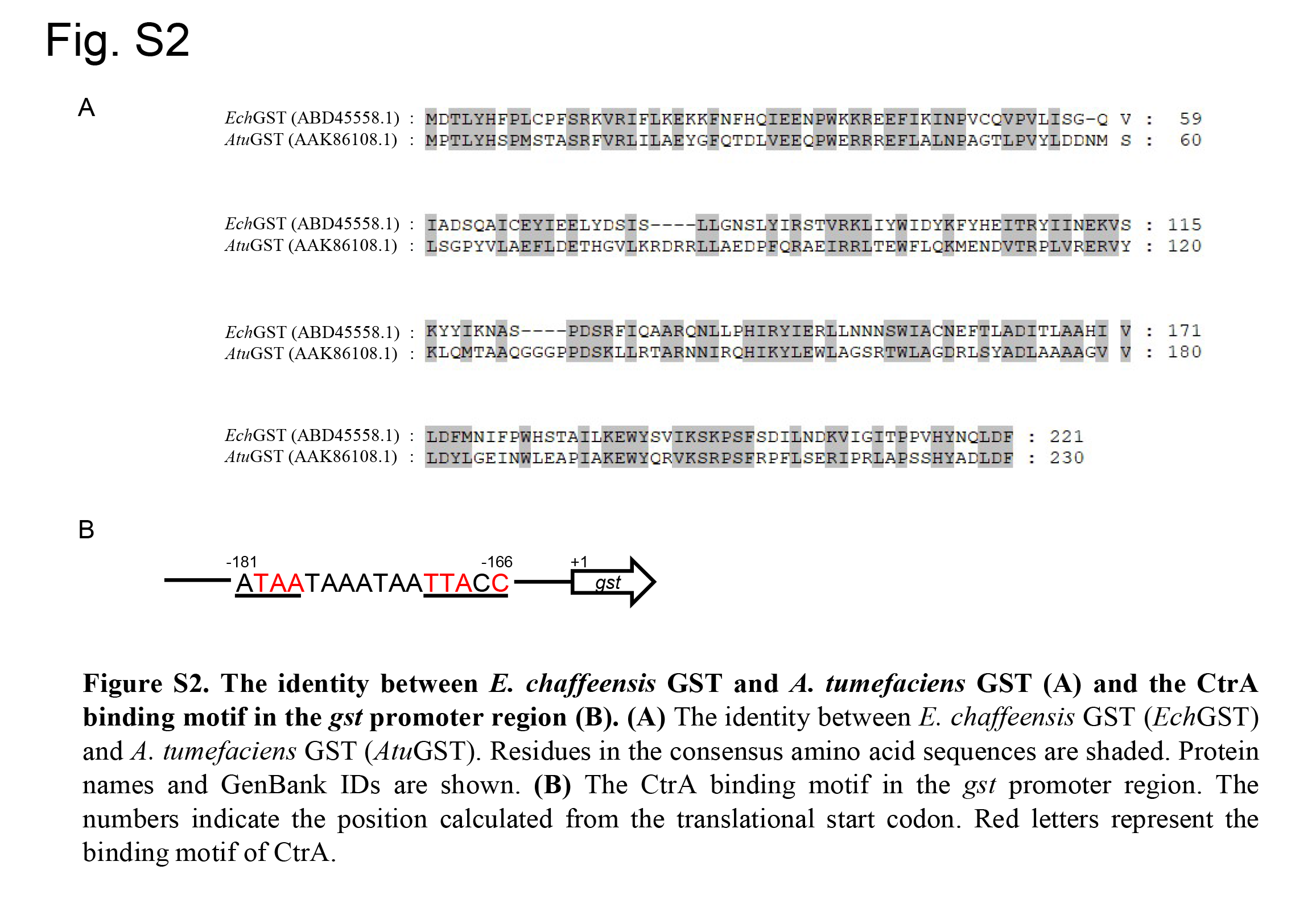

Supplement: Supplementary file 2 [file Image_2.tif]

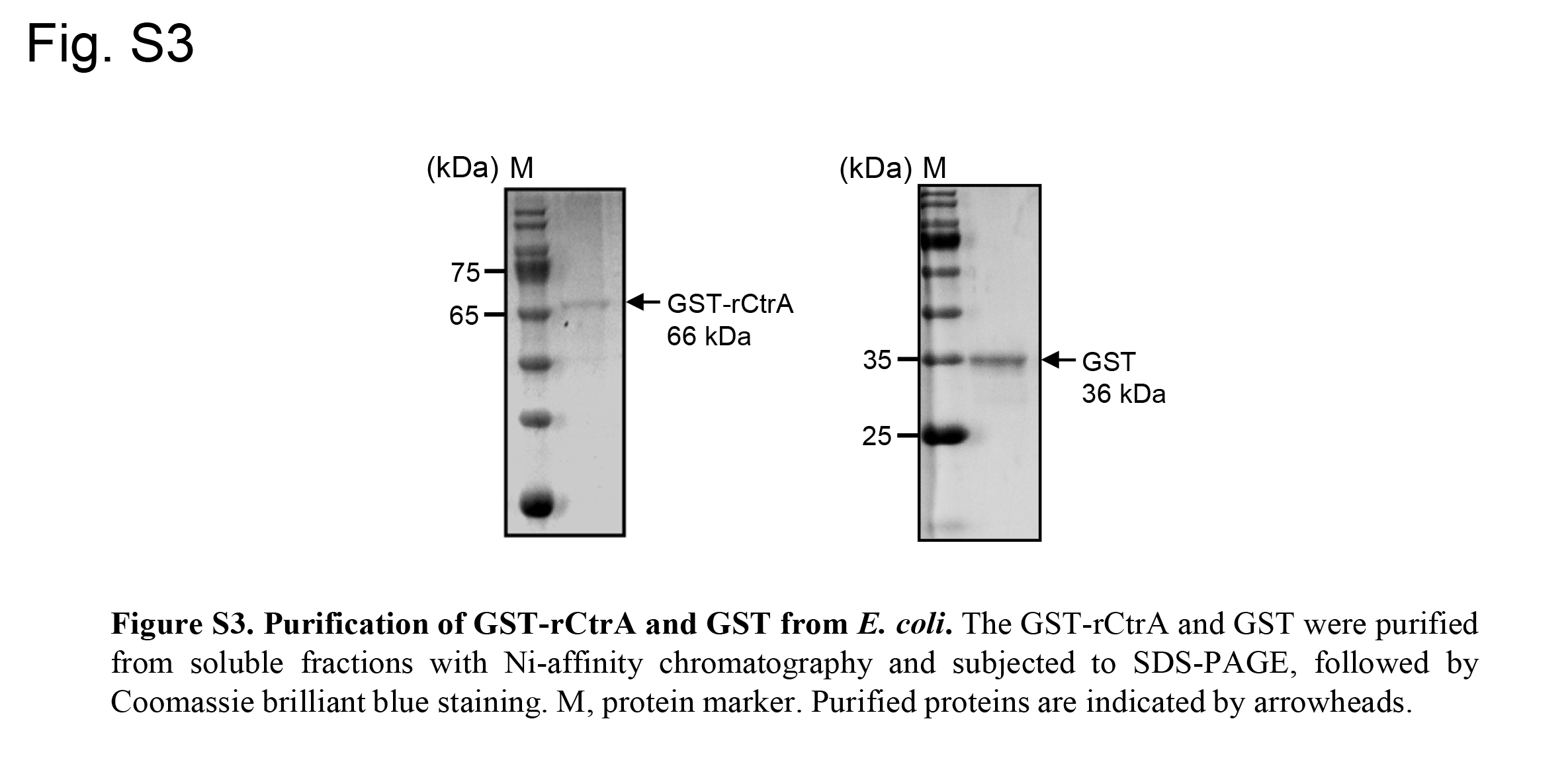

Supplement: Supplementary file 3 [file Image_3.tif]

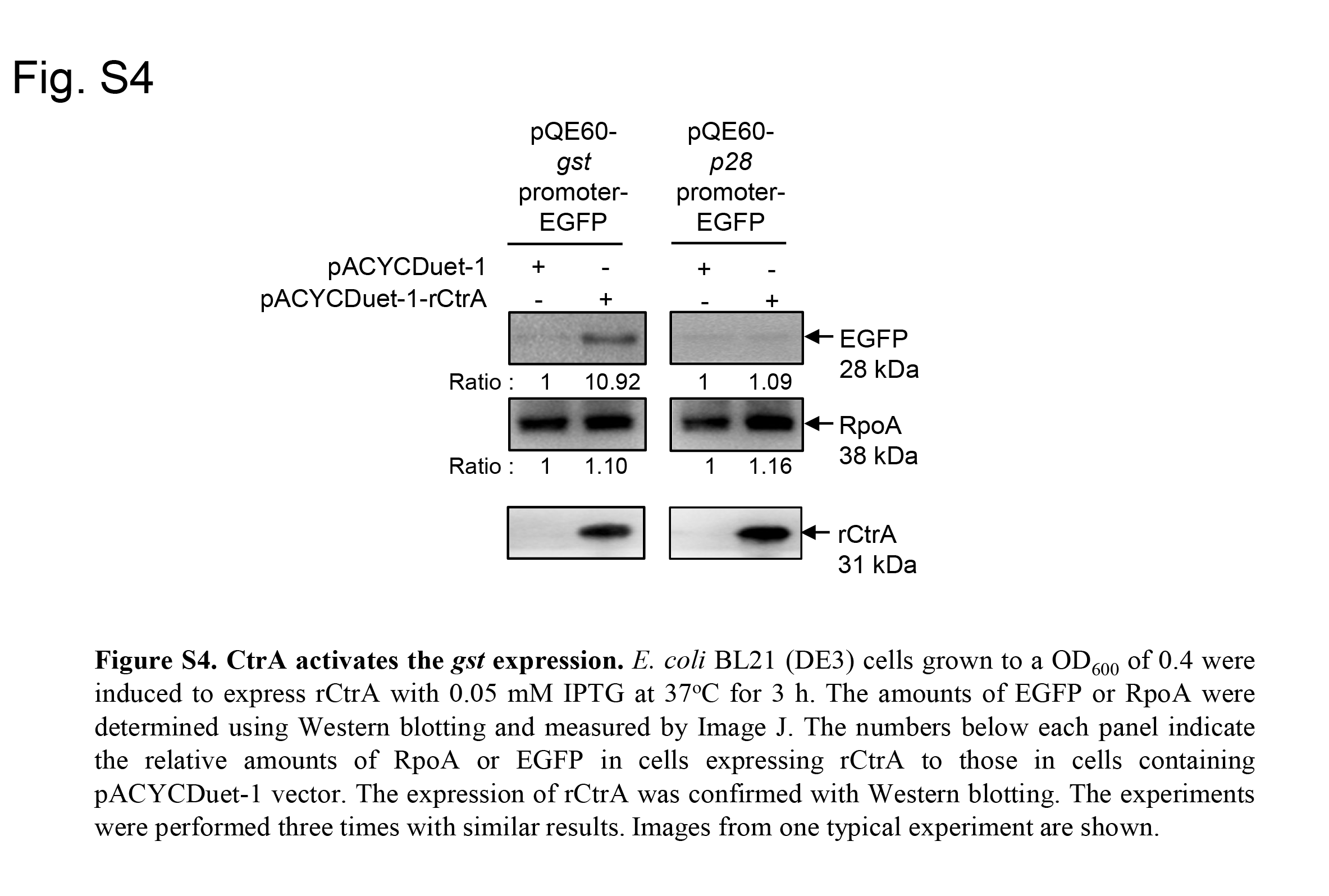

Supplement: Supplementary file 4 [file Image_4.tif]

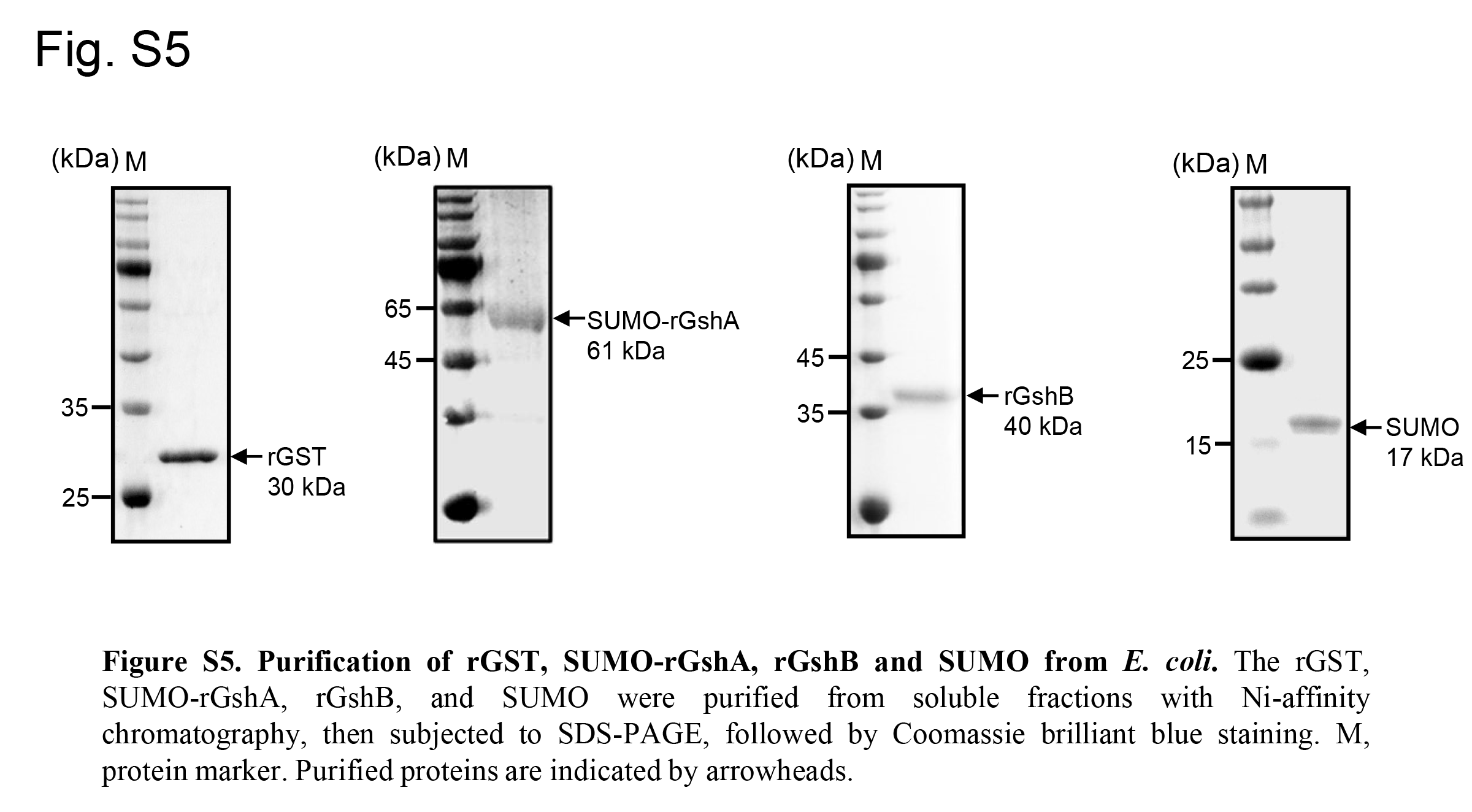

Supplement: Supplementary file 5 [file Image_5.tif]
