# Supplementary material for: CtrA activates the expression of glutathione S-transferase conferring oxidative stress resistance to Ehrlichia chaffeensis
Source: Front Cell Infect Microbiol. 2022 Dec 12;12:1081614. doi: 10.3389/fcimb.2022.1081614 (PMC9791040; doi:10.3389/fcimb.2022.1081614)
Supplement: Supplementary Table S1 — Bacterial strains, plasmids, primers, PNAs and ssRNAs used in this study. [file Table_1.docx]

**Supplementary Table S1. Bacterial strains, plasmids, primers, PNAs and ssRNAs used in this study.**

| **Strain/ Plasmid /Primer/PNA/ssRNA** | **Description** | **Source (Reference)** |
| --- | --- | --- |
| 1. ***coli* strains** | | |
| BL21 (DE3)/GST | BL21 (DE3) expressing GST; Kan^r^ | [Yan et al., 2022](#_ENREF_1) |
| BL21 (DE3)/rCtrA | BL21 (DE3) expressing GST-rCtrA; Kan^r^ | [Yan et al., 2022](#_ENREF_1) |
| BL21 (DE3)/  pACYCDuet-1 | BL21 (DE3) containing pACYCDuet-1; Chl^r^ | [Yan et al., 2022](#_ENREF_1) |
| BL21 (DE3)/  pACYCDuet-1-rCtrA | BL21 (DE3) expressing rCtrA; Chl^r^ | [Yan et al., 2022](#_ENREF_1) |
| DH5α/pQE60-*gst* promoter-EGFP | DH5α harboring pQE60-EGFP containing *gst* promoter; Amp^r^ | This study |
| DH5α/pQE60-*p28* promoter-EGFP | DH5α harboring pQE60-EGFP containing *p28* promoter; Amp^r^ | [Yan et al., 2022](#_ENREF_1) |
| BL21 (DE3)/ pACYCDuet-1/*gst* | BL21 (DE3) harboring pACYCDuet-1 and pQE60-*gst* promoter-EGFP plasmids; Chl^r^, Amp^r^ | This study |
| BL21 (DE3)/ pACYCDuet-1/*p28* | BL21 (DE3) harboring pACYCDuet-1 and pQE60-*p28* promoter-EGFP plasmids; Chl^r^, Amp^r^ | [Yan et al., 2022](#_ENREF_1) |
| BL21 (DE3)/ pACYCDuet-1-rCtrA / *gst* | BL21 (DE3) harboring pACYCDuet-1-rCtrA and pQE60-*gst* promoter-EGFP plasmids; Chl^r^, Amp^r^ | This study |
| BL21 (DE3)/ pACYCDuet-1-rCtrA /*p28* | BL21 (DE3) harboring pACYCDuet-1-rCtrA and pQE60*-p28* promoter-EGFP plasmids; Chl^r^, Amp^r^ | [Yan et al., 2022](#_ENREF_1) |
| BL21 (DE3)/pET-33b(+)-rGST | BL21 (DE3) expressing rGST; Kan^r^ | This study |
| BL21 (DE3)/pET-33b(+)-rGshB | BL21 (DE3) expressing rGshB; Kan^r^ | This study |
| BL21 (DE3)/pET-His-SUMO | BL21 (DE3) expressing SUMO; Kan^r^ | This study |
| BL21 (DE3)/pET-His-SUMO-rGshA | BL21 (DE3) expressing SUMO-rGshA; Kan^r^ | This study |
| **Plasmid** |  |  |
| pET-41a(+) | Clone vector; Kan^r^ | Novagen |
| pCtrA | pET-41a(+) harboring *ctrA* gene; Kan^r^ | [Yan et al., 2022](#_ENREF_1) |
| pACYCDuet-1 | Clone vector; Chl^r^ | Novagen |
| pACYCDuet-1-rCtrA | pACYCDuet-1 harboring *ctrA* gene; Chl^r^ | [Yan et al., 2022](#_ENREF_1) |
| pQE60-EGFP | Amp^r^ | From Doctor Bi |
| pQE60-*gst* promoter-EGFP | pQE60-EGFP harboring *gst* promoter; Amp^r^ | This study |
| pQE60-*p28* promoter-EGFP | pQE60-EGFP harboring *p28* promoter; Amp^r^ | [Yan et al., 2022](#_ENREF_1) |
| pET-33b(+) | Clone vector; Kan^r^ | Novagen |
| pET-33b(+)-rGST | pET33b(+) harboring *gst* coding sequence; Kan^r^ | This study |
| pET-33b(+)-rGshB | pET33b(+) harboring *gshB* coding sequence; Kan^r^ | This study |
| pET-His-SUMO | Clone vector; Kan^r^ | Lab stock |
| pET-His-SUMO-rGshA | pET-His-SUMO harboring *gshA* coding sequence; Kan^r^ | This study |
| **Primer** | **Sequence (5’→3’)** | **Function** |
| CtrA-F | GTCCCATGGGAATGCGTATATTATTAATAGAAGATG | Protein expression |
| CtrA-R | GGCTCGAGTTATGCTTCCTCAACATACTTTTTA | Protein expression |
| GshA-F | CAGAATTCATGACGGTAATTATTGATACATTAAATG | Protein expression |
| GshA-R | GCAAGCTTTTAACTTAGTAAGCAATTTTGCTCC | Protein expression |
| GshB-F | CCGAATTCGATGGCACTAATTGTTGCTTTTC | Protein expression |
| GshB-R | TGCTCGAGCTATATACTATTATGTTTTTCATAAAACTTCTCTTC | Protein expression |
| GST-F | CGGGATCCGATGAAATTTACTATGGATACATTGTATCATTTTCCAC | Protein expression |
| GST-R | GCAAGCTTTTAGAAATCCAGTTGATTGTAATGTACAGGAGG | Protein expression |
| pACYCDuet-1-rCtrA-F | GGGAGATCTCATGCGTATATTATTAATAGAAGA | EGFP reporter assay |
| pACYCDuet-1-rCtrA-R | CGGGGTACCTTATGCTTCCTCAACATACTTT | EGFP reporter assay |
| pQE60-*gst* promoter*-*F | GTGCTCGAGTTTCTAGGC | EGFP reporter assay |
| pQE60-*gst* promoter*-*R | ATGAATTCCAGACTTGGTAACCTTGTGC | EGFP reporter assay |
| pQE60-*p28* promoter-F | GGGCTCGAGtgctgcaggtaaataaaaatagt | EGFP reporter assay |
| pQE60-*p28* promoter*-*R | CCCGAATTCATATAACCTAATAGTGACAAATAAA | EGFP reporter assay |
| *gst-*promoter*-*F | GTGCTCGAGTTTCTAGGC | EMSA |
| *gst-*promoter*-*R | CAGACTTGGTAACCTTGTGC | EMSA |
| *p28-*promoter-F | TGCTGCAGGTAAATAAAAATAGT | EMSA |
| *p28-*promoter*-*R | ATATAACCTAATAGTGACAAATAAA | EMSA |
| *16S rRNA*-F  (*E. chaffeensis*) | GGTGAGTAATGCGTAGGAATC | qRT-PCR |
| *16S rRNA*-R  (*E. chaffeensis*) | GCTCATCTAATAGCGATAAATC | qRT-PCR |
| *gshA-*qRT-F | CGGTTGATTTAAGAGTATCTGA | qRT-PCR |
| *gshA-*qRT-R | GCTACAGTTCGCATAATTAACTA | qRT-PCR |
| *gshB*-qRT-F | ATTGCCAGAGCATGCTTGGTA | qRT-PCR |
| *gshB*-qRT-R | CAAATGGTGGGTTTTGACGCA | qRT-PCR |
| *gst*-qRT-F | GCAGATAGCCAAGCAATTTGTGAATAC | qRT-PCR |
| *gst*-qRT-R | GCACTGTTGACCTTATGTATAGTGAATTCC | qRT-PCR |
| **PNA** | **Sequence (5’ →3’)** | **Function** |
| CtrA | CTGCCTTTGCACATGC | CtrA knockdown |
| GshA | TATCATTTAATGTATC | GshA knockdown |
| GshB | CATCTTTATCCATTTG | GshB knockdown |
| GST | TACAATGTATCCAT | GST knockdown |
| Control (CTL) | GGCTCTATACAC | Negative control |
| **ssRNA** | **Sequence (5’ →3’)** |  |
| GshA | AAUUAUUGAUACAUUAAAUGAUAUAUUAACAA | Hybridization assay |
| GshB | UGCUUUUCAAAUGGAUAAAGAUGUUGUUGUUG | Hybridization assay |
| GST | AUUUACUAUGGAUACAUUGUAUCAUUUUCCA | Hybridization assay |

The enzymes sites are indicated by the underline.

The complementary bases to corresponding PNA are indicated by red letters.

Kan^r^, kanamycin resistance; Amp^r^, ampicillin resistance; Chl^r^, chloramphenicol resistance.

REFERENCES：

Yan, J., Liang, Q., Chai, Z., Duan, N., Li, X., Liu, Y., et al. (2022). Glutathione synthesis regulated by CtrA protects *Ehrlichia chaffeensis* from host cell oxidative stress. *Front Microbiol* 13**,** 846488. doi: 10.3389/fmicb.2022.846488.
